# Experimental Study of Stress and Deformation of Reclaimed Asphalt Concrete at Different Temperatures

**DOI:** 10.3390/ma16031323

**Published:** 2023-02-03

**Authors:** Jing Zhang, Mingyuan Zhou, Juan Liu, Xianwen Huang

**Affiliations:** 1 College of Architecture and Civil Engineering, Yancheng Polytechnic College, Yancheng 224005, China; 2 School of Civil Engineering, Jiangsu University of Science and Technology, Zhenjiang 212003, China; 3 Zhenjiang Xinbo Consulting Co., LTD, Zhenjiang 212000, China; 4 School of Civil Engineering, Suzhou University of Science and Technology, Suzhou 215009, China

**Keywords:** recycle asphalt concrete, stress and strain, triaxial test, constitutive model

## Abstract

Asphalt concrete has been used as a material for dam core walls because of its impermeability, durability and reliability. Firstly, asphalt is a temperature-sensitive material, and many of its characteristics are related to temperature. Secondly, because of the increasing construction height of the dam, the pressure on the asphalt concrete core wall is also great. Finally, for the purpose of resource utilization, it is necessary to verify whether the reclaimed asphalt concrete can be used in dam construction. Therefore, it is necessary to study the stress and deformation characteristics of recycled asphalt concrete under different temperatures and confining pressures. In this study, three groups of triaxial tests of reclaimed asphalt concrete were carried out for the first time in a new temperature-controlled room. Duncan Zhang’s E-v model was used to fit the test results. The results show that the stress–strain curves of reclaimed asphalt concrete show softening characteristics at low temperatures and low confining pressure. It evolves to a hardening type with the increase in temperature and confining pressure. The bulk curve is first contracts but is followed by dilatancy. The dilatancy characteristics become more obvious at low temperatures and low confining pressure. With the increase in temperature and confining pressure, the dilatancy characteristics will weaken. Duncan Zhang’s E-v model has a good fitting effect on the stress–strain relationship but a poor fitting effect on the volumetric curve. The research of this paper can better combine the utilization of waste resources with engineering and achieve the aim of resource-saving and waste utilization under the premise of ensuring the safety of the engineering

## 1. Introduction

Asphalt concrete is a very good engineering material. Because of its impervious deformation and coordination characteristics, it is not only used in industry, agriculture and other fields but also widely used in roads, airports and water conservancy projects. Especially with the rapid development of water conservancy constructions in recent years, asphalt concrete is widely used in the seepage control of dams, and a series of asphalt concrete core wall dams are constructed [1,2,3,4,5]. Many scholars [6,7,8,9,10,11] have conducted a lot of research on its strength and deformation characteristics. Asphalt concrete is a typical temperature-sensitive material, and its mechanical properties are greatly affected by temperature. Wei Yun [12] studied the asphalt concrete’s mechanical properties with the variation of temperature using the triaxial test. Based on Duncan Zhang’s model, the variation rule of the model parameters with temperature is studied. The fitting degree of the model under different temperatures is analyzed with the statistics. Wang Jing-wen [13] studied the mechanical properties of asphalt concrete at 0~20 °C with the triaxial compression test and triaxial creep test, finding different effects on the material properties of the temperature. Wang Liang [14] established a three-dimensional aggregate model according to the aggregate gradation in asphalt concrete specimens and used this model to conduct a discrete element study on uniaxial compression tests under different strain rates. Chen Yu [15] conducted two kinds of asphalt concrete tests, respectively, at different temperatures by indoor high pressure and a temperature control triaxial instrument to study the influence of temperature on its mechanical properties.

In addition, in order to achieve the purpose of recycling waste resources, many scholars have conducted a lot of research on the performance of recycled asphalt concrete. Liu Song an [16] studied recycled asphalt concrete’s design method of mix ratio, road performance and durability modified with composite fiber and natural polymer asphalt. Zhou Zhi gang [17] proposed an optimal design method for recycled asphalt that comprehensively considers the requirements of technical performance and economic benefit index of reclaimed asphalt through the study of plant heat mix and SBS-modified asphalt pavement engineering. Hu Ya chun [18] proposed a research idea of improving the strength of cold-reclaimed asphalt concrete by using a water-based environment-friendly asphalt pavement regenerating agent. Wang Hai feng [19] studied the adaptability between cement-fly ash and recycled aggregate systems based on the recovery of asphalt pavement materials. Bian Hai ning [20] studied and discussed the mixed design of recycled asphalt concrete for old pavement. Using the recycling technology of discarded pavement materials to achieve the goal of saving production costs and reducing energy consumption, Li Cai hui [21] conducted a splitting test and compression test on a cold-reclaimed asphalt mixture with different used material contents to determine the reasonable mixing amount of light oil regenerated agents under different used material contents. Then, a water stability test, high-temperature stability test and low-temperature anti-cracking performance test were conducted on a cold-reclaimed asphalt mixture with a reasonably regenerated agent to study the mixing amount of used material and the cold-reclaimed asphalt with light oil regenerated agent’s relationship to the road performance of mixtures.

Sumeda Paranavithana [22] found that recycled concrete aggregates (RCA), which are produced by crushing demolished concrete elements, differ from fresh aggregates due to the cement paste attached to the surface of the original natural aggregates after the process of recycling. This highly porous cement paste and other contaminations contribute to the lower particle density and higher porosity variation in the quality of the RCA and higher water absorption. It was also found that all the volumetric properties (except the percentage of air voids), resilient modulus and creep values of asphalt specimens containing RCA as coarse aggregates were relatively lower compared with the values found for similar specimens made with only fresh aggregates. Xun hao Ding [23] investigated the performance of recycled asphalt concrete with stable crumb rubber asphalt (SCRA) binder. Both the normal recycled asphalt mixtures and the SCRA recycled asphalt mixtures were prepared with 0, 30 and 50% content of the reclaimed asphalt pavement (RAP). Laboratory tests were carried out to compare the performance of different mixtures, and the comprehensive recycling effect of the SCRA and rejuvenator were evaluated further. The SCRA is much better than virgin asphalt (VA) in recycling aged asphalt mixtures with large RAP content, which could reach 50%. The rejuvenator has a positive effect on low-temperature performance, moisture stability and fatigue resistance. By combining the use of the SCRA and rejuvenator, the low-temperature performance of the SCRA recycled asphalt mixtures could be further improved while the other performance retains a high level. Jie Ji [24] studied the recycled asphalt concrete (RAC) fatigue properties test plan, and the influence of RAs on it was analyzed, finding that it ignores the applicability of different test methods and the impacts of regional temperature and climate conditions. Future research directions and recommendations for a test plan regarding the fatigue properties of RAC are proposed. Research should focus on the effect of RA properties on fatigue properties. It should be carried out with reasonable test methods, service life characterization models, and climate-identical test conditions to clarify the durability index. The evaluation system of fatigue properties of RAC should be established, and solid theoretical supports for the application of construction and demolition wastes (CDWs) should be provided, thereby accelerating the application of recycled asphalt pavement containing CDWs.

Chen, J.S [25] used three reclaimed asphalt concrete (RAC) stockpiles sampled, and the aged binders recovered from RAC binders were mixed with recycling agents at ten levels to produce bitumen blends. The blends using virgin bitumen as the softening agent exhibited a significantly different rheological behavior from the ones using the rejuvenating agent. The addition of a recycling agent could shift up or down the master curve of the blend, depending on the engineering properties of the recycling agent. A normalized viscosity ratio (NVR) model was used to characterize the rheological properties of aged bitumen mixed with softening and rejuvenating agents. An interaction parameter was introduced into the model to consider the physico-chemical reaction between aged bitumen and the recycling agent. This mixing rule was compared to the method specified in the blending chart by the Asphalt Institute (AI). The blending chart was shown to be applicable to determine the amount of the softening agent required to meet the target viscosity. The NVR model appeared to be a better tool for the rejuvenating agent to predict the viscosity of a recovered bitumen blend than the AI chart. 

M. R studied [26] a total of four different mixtures prepared by partially replacing natural aggregates with recycled asphalt concrete (RAC) in different proportions. The results showed that adding RAP to the mixture reduced the mechanical strength of the roller-compacted concrete (RCC) mixture. The best mechanical strength was obtained at 50% RAC replacement. To increase the mechanical strength of RCC made with 50% RAP, Micro Silica (MS) with partial replacement of cement at 3, 6, 9 and 12% was used. Increasing the MS content from 3 to 12% caused the mechanical strength of all mixtures to increase. The results [27,28,29] showed that RCC with 50% RAC and 9% MS had about 20% more compressive strength. Moreover, the increase in tensile splitting and flexural strength was 6 and 2%, respectively. The statistical analysis indicates that there was a strong relationship (R2 > 0.98) between flexural strength values estimated from the regression model and the measured laboratory values (this relationship was about 0.84 for compressive strength and tensile splitting strength). The estimation of tensile splitting strength and flexural strength with respect to compressive strength showed that there was a strong (R2 > 0.98) and good relationship (R2 > 0.75) between them, respectively.

However, there are a few pieces of research on regenerating old asphalt pavement concrete that were applied to the dam core wall. Reusing waste has always been the goal of our human society to seek sustainable development; based on this purpose, studying the performance of recycled asphalt concrete is very necessary. In order to study the influence of temperature and confine pressure on the characteristics of recycled asphalt concrete further, three groups of triaxial tests under different temperatures and confining pressure were carried out in this paper, and then the test results were fit with Duncan Zhang’s E-v hyperbolic model.

## 2. Materials and Methods

### 2.1. Test Facility

**Figure 1:**
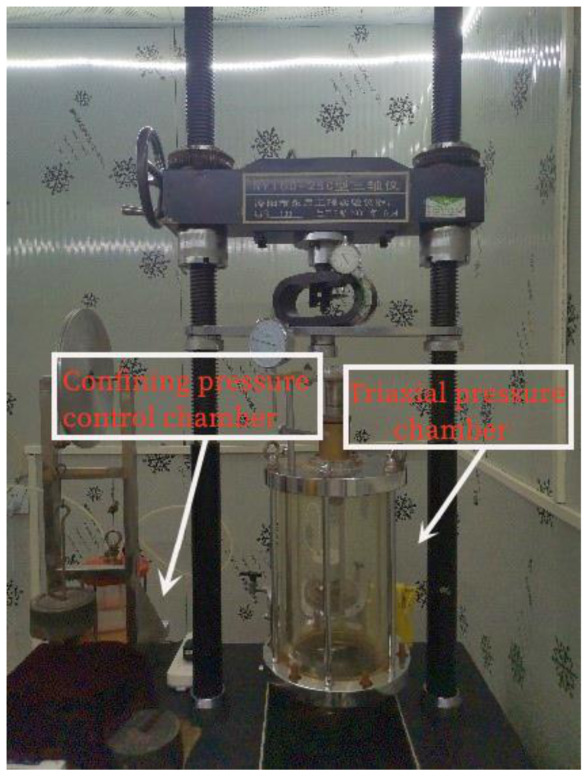
SY100-25 °C temperature-controlled triaxial test instrument.

The triaxial test of recycled asphalt concrete was carried out on SY-250 (Jiang Su Yong Chang, Jiangsu, China) as shown in Figure 1. The sample size of this test is Φ101 mm × 200 mm. Strain control was adopted in the test. The test speed was at rate of 0.5 mm/min, and the test was stopped when the axial strain reached 25%. Stress and strain during the test are reflected by dial gauge and ring readings. Considering the pressure on the asphalt concrete core wall, four confining pressures were set at 0.2, 0.5, 0.8 and 1.1 MPa, respectively. During the test, the confining pressure is provided by a high-pressure gas cylinder, which is connected to the electric air compressor. This ensures reliable and stable pressure during the test. The volume variation in the test is measured by measuring weight of confining pressure control chamber. When the volume of the sample expands, the water in the triaxial pressure chamber is discharged to the confining pressure control chamber, and the weight of the confining pressure control chamber increases. On the contrary, when the volume of the sample shrinks, the water in the confining pressure control chamber is sucked into the triaxial pressure chamber. The confining pressure control chamber weight is reduced. The volume change is converted by mass transformation of the confining pressure control chamber. The biggest difference between this temperature control triaxial instrument and the previous one is that the triaxial instrument is placed in a room enclosed by thermal insulation materials. The kind of room can play a role in isolating the external environment temperature. It also has the effect of heat insulation to meet the temperature required by the test. The required temperature is adjusted by air conditioning before the 8 h of the test. Therefore, the ambient temperature can be maintained within the allowable error range of the test time. It can better study the influence of temperature change on the results of the triaxial test.

### 2.2. Test Sample Preparation

The asphaltic concrete in this test was taken from the old pavement. The asphalt concrete was heated, and then the regeneration agent was added and then was compacted. The compaction hammer with a weight of 4536 g, and the height of asphaltic compaction concrete is 457 mm. First, the asphalt material was heated to 130 °C, and the mixing pot was heated to 150 °C; then, the asphalt was added and mixed in the mixing pot for 1 min, and the regeneration agent was added and mixed again for 2 min. The discharge temperature was 140 ± 5 °C. The sample is compacted into 3 layers, 110 times for each layer. Then, the test samples cooled for 24 h together with the mold. The samples were placed in room for seven days. Before the test, the samples were placed in a thermostatic water bath, which was adjusted to the temperature of the test. After curing for 24 h, the triaxial test on was carried out. In the whole test process, the temperature change of the enclosed space did not exceed ±0.5 °C. The test equipment and scheme are shown in Figure 1 and Table 1, respectively.

## 3. Results

### 3.1. Stress–strain Relation Curve

**Figure 2:**
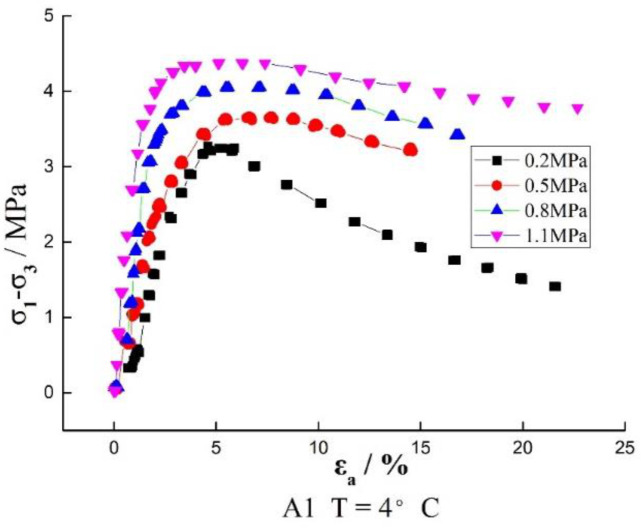

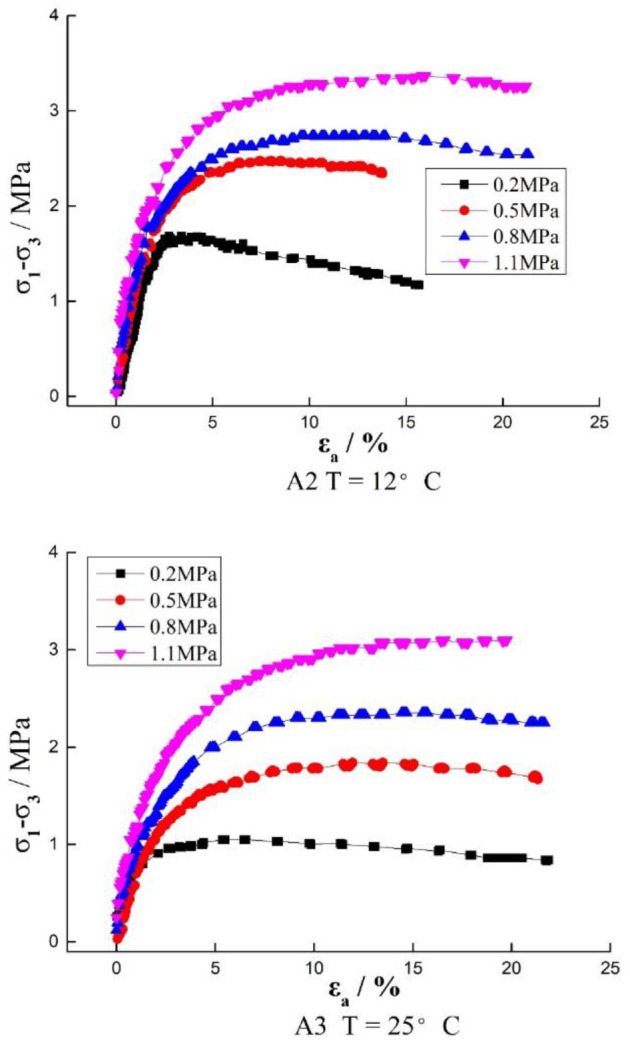
Stress–strain relation curves at different temperatures.

The stress–strain curves of the three groups of samples obtained by the large strain triaxial tests are shown in Figure 2. As we can see from Figure 2, the stress–strain curves of recycled asphalt concrete at different temperatures have two types: hardening and softening. At the low temperature of 4 °C, the curves basically show a softening type. The lower the confining pressure is, the more the softening effect is obvious. The stress drops quickly after softening. When the confining pressure is 0.2 MPa, the softening stress decreases to half of the peak stress at the final strain, which is obviously different from that of other brittle materials. It is clear that the asphalt played a role in the damage. However, with the increase of confining pressure, the curve gradually has a trend of softening to hardening. At the temperature of 12 °C, when the confining pressure is 0.2 MPa, the curve shows an obvious softening type. When the confining pressure increases to 0.5, 0.8 and 1.1 MPa, the softening effect becomes not obvious, and it turns into a hardening type when it reaches 1.1 MPa. At the temperature of 25 °C, in addition to the curve of 0.2 MPa, the curves are all hardening types basically. The stress keeps increasing with the increase of strain. When the strain reaches 10%, the stress basically remains unchanged. In summary, it can be seen that the stress–strain curves of reclaimed asphalt concrete are closely related to temperature and confining pressure. Under the condition of low temperature and low confining pressure, it presents a softening type. With the increase in temperature and confining pressure, the softening type gradually evolves into the hardening type.

### 3.2. Volume Change Relation Curve

**Figure 3:**
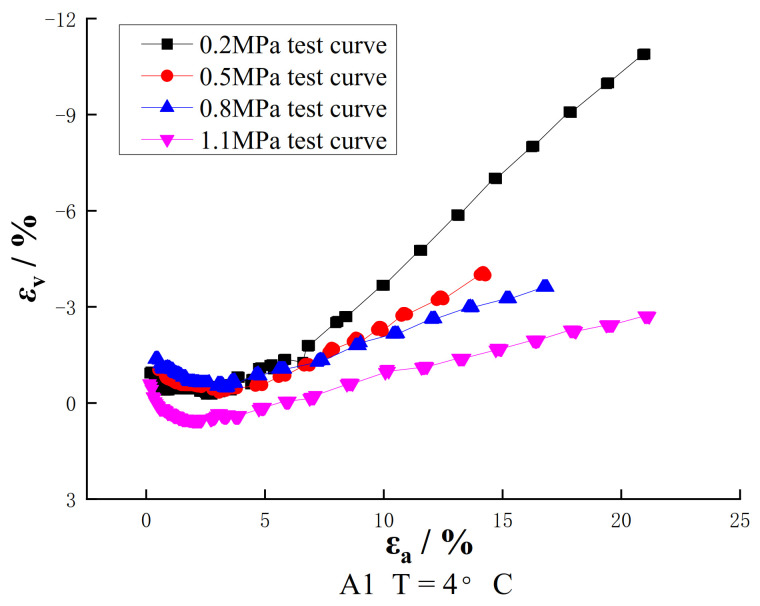

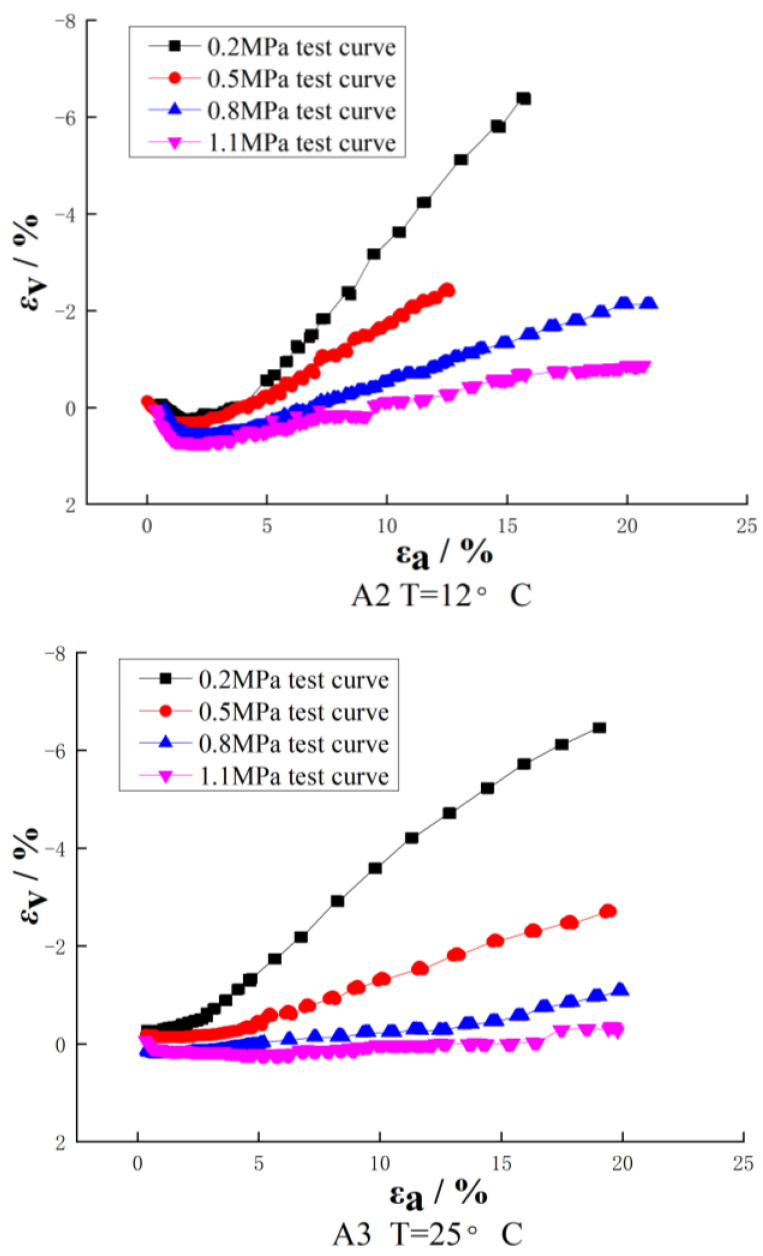
Volume change relation curve.

The volume variation curves of the three groups of samples obtained by the large strain triaxial test are shown in Figure 3. As we can see from Figure 3, under different temperatures and confining pressures, the volumetric variation curves of reclaimed asphalt concrete basically start with shear contraction but are followed by dilatancy. It shows a state of shear contraction before the axial strain $\varepsilon_{a}$ reaches 3% and then changes to dilatancy.

At the low temperature of 4 °C and confining pressure of 0.2 MPa, the dilatancy rate can reach 12% with the increase of strain. Combined with the stress–strain curve, it can be seen that the dilatancy occurs before the peak stress, which indicates that the internal particles need to roll over another part of the particles and produce relative dislocation, which requires greater bite force and is manifested as higher stress. With the increase of strain, one part of the particle bypasses another part of the particle, the structure becomes loose, the stress is reduced, and therefore, the performance is softened. As a whole, the lower the temperature, the smaller the confining pressure and the more severe the dilatancy; with the increase in temperature and confining pressure, the dilatancy effect will be weaker and weaker. It shows that the deformation of recycled asphalt concrete is also closely related to temperature and confining pressure.

Shear Strength Parameter Curve

**Figure 4:**
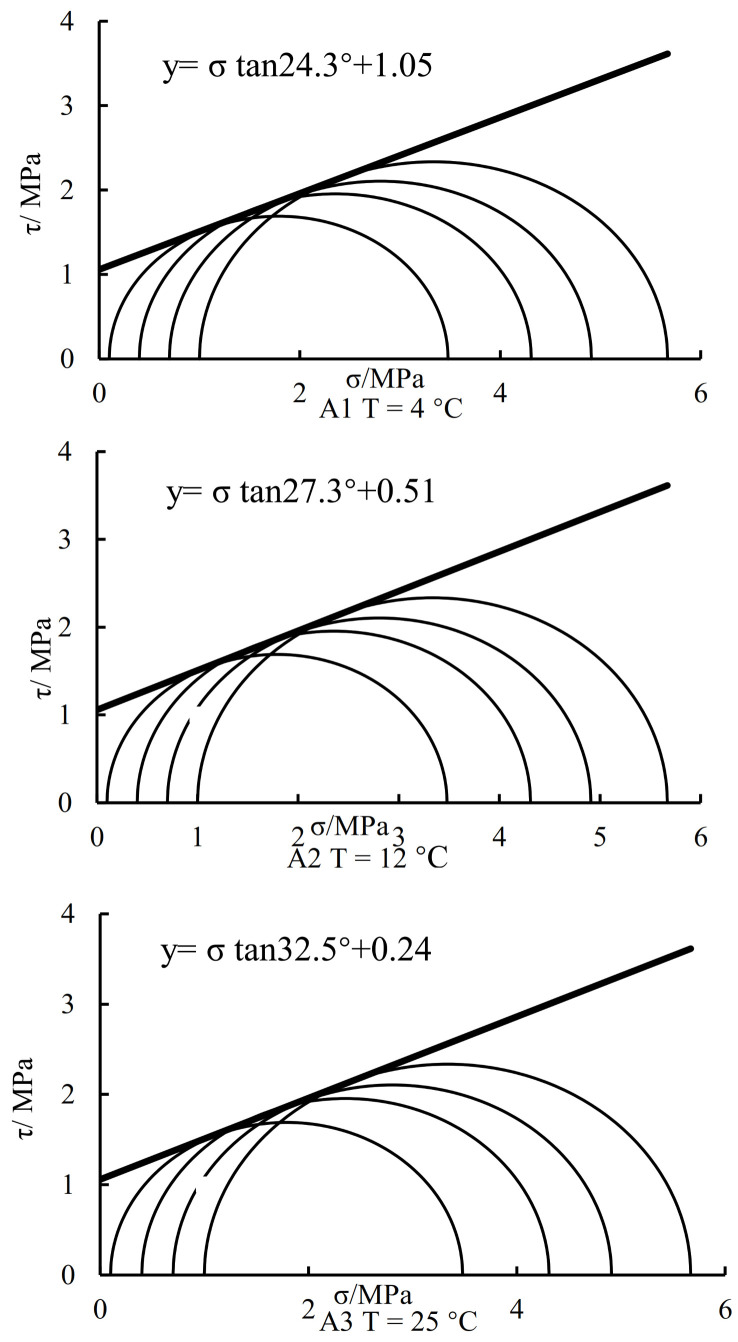
Shear strength parameter curve.

According to the peak deviatoric stress of recycled asphalt concrete at different temperatures, the molar coulomb circle was drawn, and the shear strength parameters were fitted. As can be seen from Figure 4, the shear strength parameters of reclaimed asphalt concrete at different temperatures are different. With the increase in temperature, the value of c decreases while the value of φ increases. The higher of temperature is, the smaller of c is and the larger of φ. This indicates that the shear strength of recycled asphalt concrete is temperature dependent, which requires special attention. The temperature increase has a great influence on the internal friction angle, which makes the reclaimed asphalt concrete show different shear characteristics at different temperatures, and it has a great influence on the dam core wall constructed by it.

## 4. Discussion

### 4.1. Introduction of Duncan Zhang’s E-v Hyperbolic Model

Duncan Zhang E-ν model $E_{t}$; Poisson’s ratio $\nu_{t}$; Stress level failure ratio $R_{f}$; stress level S; Initial Poisson’s ratio $\nu_{i}$ and A are calculated as follows:

(1) $$E_{t}={\left(1-R_{f}S\right)}^{{}^{2}}Kp_{a}{\left(\frac{\sigma_{3}}{p_{{}_{a}}}\right)}^{n}$$

(2) $$\nu_{t}=\frac{\nu_{i}}{{\left(1-A\right)}^{2}}$$

(3) $$R_{f}=\frac{{\left(\sigma_{1}-\sigma_{3}\right)}_{f}}{{\left(\sigma_{1}-\sigma_{3}\right)}_{u}}$$

(4) $$S=\frac{\left(\sigma_{1}-\sigma_{3}\right)}{{\left(\sigma_{1}-\sigma_{3}\right)}_{f}}=\frac{\left(\sigma_{1}-\sigma_{3}\right)\left(1-\sin\varphi \right)}{2c\cos\varphi +2\sigma_{3}\sin\varphi }$$

(5) $$\nu_{i}=G-F\mathrm{lg}\frac{\sigma_{3}}{p_{a}}$$

(6) $$A=\frac{(\sigma_{1}-\sigma_{3})D}{Kp_{a}{\left(\frac{\sigma_{3}}{p_{a}}\right)}^{n}(1-R_{f}S)}$$

In the formulas, ${\left(\sigma_{1}-\sigma_{3}\right)}_{f}$ is the maximum value of the deviatoric stress test; ${\left(\sigma_{1}-\sigma_{3}\right)}_{u}$ is the extreme asymptotic value of deviatoric stress; $p_{a}$ is the atmospheric pressure; c is cohesive force; φ is the angle of internal friction; D*,* F*,* G*,* K*,* n are the model parameter.

Duncan Zhang’s E-v model parameters of the three groups of recycled asphalt concrete materials determined according to the above calculation formula are shown in Table 2.

### 4.2. The Results of Stress–Strain Fit of Duncan Zhang’s E-v Model Are Compared with Experimental Results

**Figure 5:**
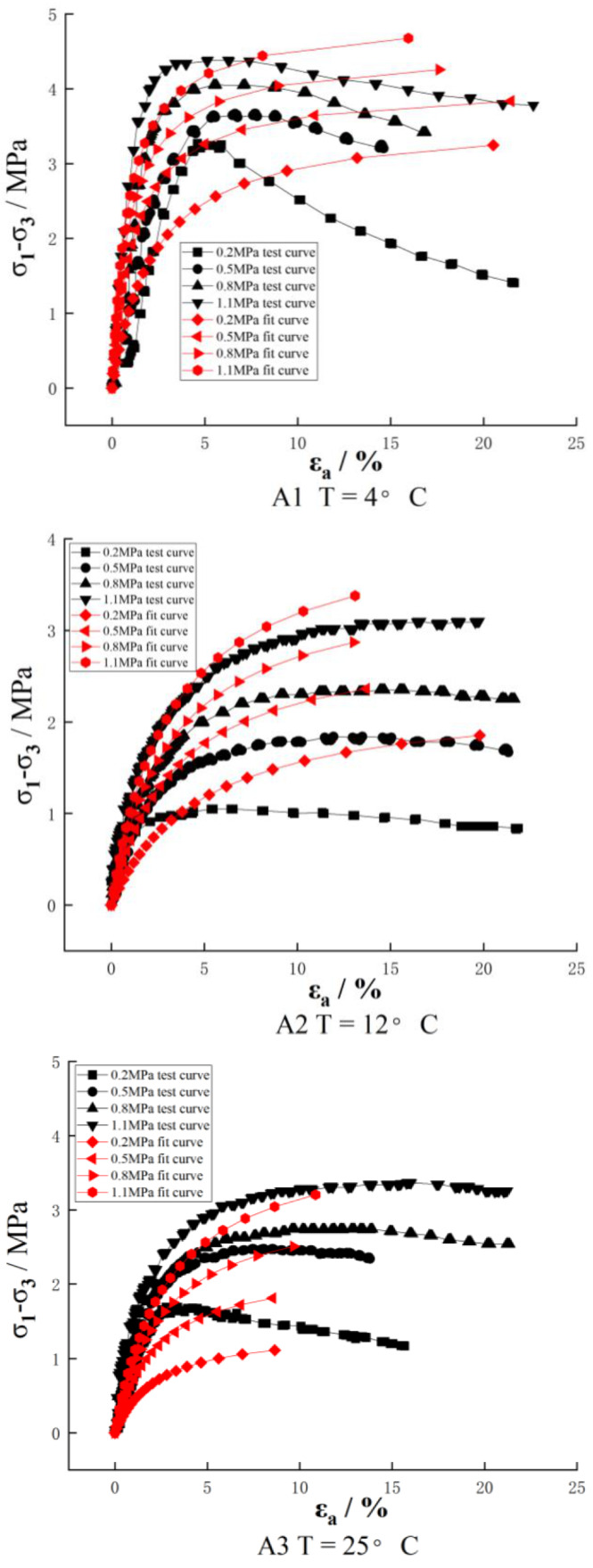
The fitting results are compared with the experimental results.

The stress–strain curves of reclaimed asphalt concrete under different temperatures and confining pressure can be fitted by Duncan Zhang’s E-v model parameters. According to Figure 5, at the low temperature of 4 °C and before the peak point of the test curve, the rest of the fitting curve and the test curve have a certain degree of fit. However, after the peak point, the stress value decreases with the development of the strain since Duncan Zhang’s model is a hardened curve. With the development of the strain, the stress value increases continuously. At 12 °C, all the test curves showed a hardening type. At 25 °C, in addition to the curve of 0.2 MPa, the test curves were all hardened. 

In summary, it can be seen that recycled asphalt concrete has softening characteristics under low confining pressure, and Duncan Zhang’s E-v model is a hardening model, so the stress–strain relationship fitting effect of the reclaimed asphalt concrete under low confining pressure is poor, and the softening phenomenon after the peak cannot be fitted. However, it has a certain applicability under high confining pressure.

### 4.3. Comparison of Duncan Zhang’s E-v Model with Experimental Results of Volume Variation

**Figure 6:**
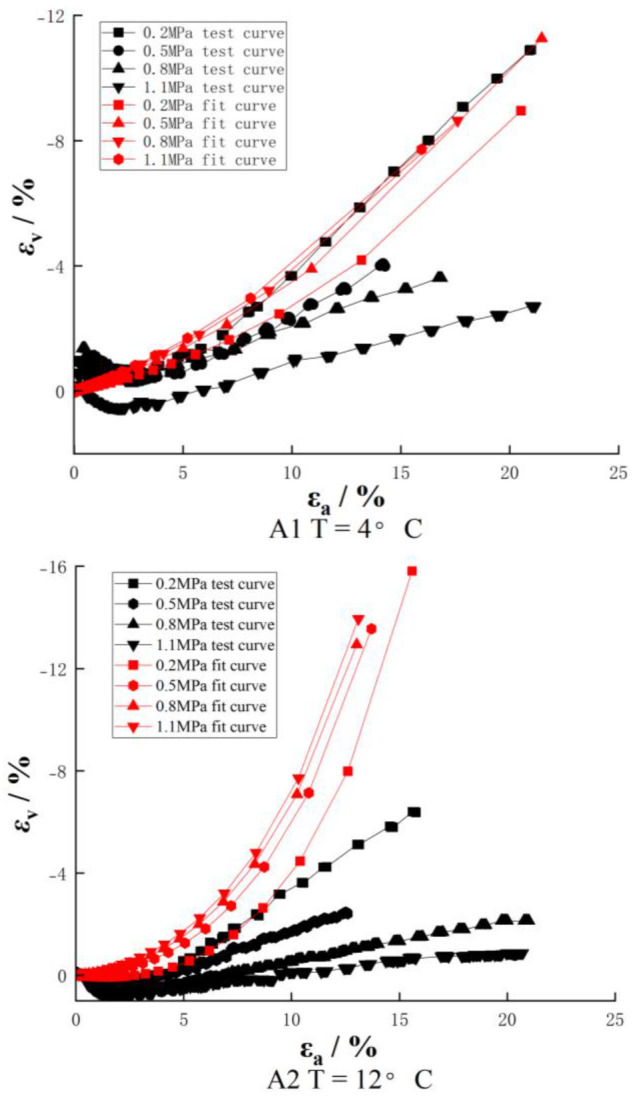

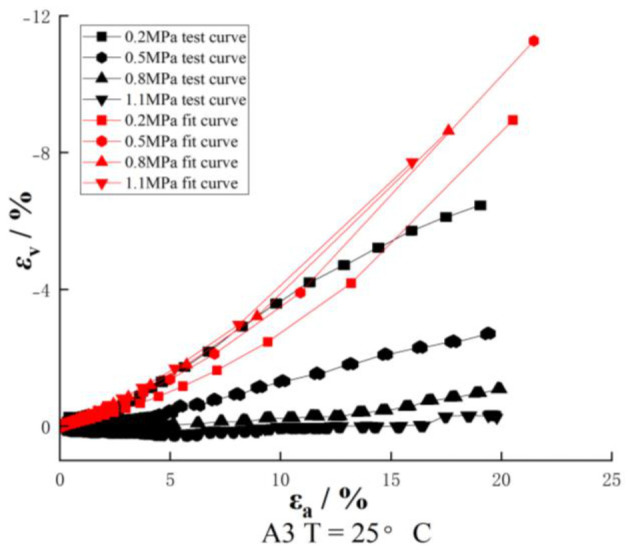
The fitting results are compared with the experimental results.

The volume curves of reclaimed asphalt concrete under different temperatures and confining pressures can be fitted by Duncan Zhang’s E-v model parameters. It can be seen from Figure 6 that test curves at different temperatures are basically shear contraction but followed by dilatation, while the fitted curves are all dilatation. Under the same strain, for test curves, the larger the confining pressure, the larger the dilatancy. However, different from Poisson’s ratio of Duncan Zhang’s E-v model, the larger the confining pressure, the smaller the test measured Poisson’s ratio. The test measured Poisson’s ratio increases with the stress level in a positive correlation too.

There is a poor fit effect between the fitting curves and test curves at 4 °C. However, there is a large deviation between the fitting curves and the test curves at the other two temperatures. This indicates that Duncan Zhang’s E-v model can represent the dilatancy characteristics of materials, but its fitting effect on the experimental results is poor. In addition, when using Duncan Zhang’s E-v model to fitting dilatancy, all Poisson’s ratios are greater than 0.5. It can be seen that the fitting effect of Duncan Zhang’s E-v model on the volume variation of reclaimed asphalt concrete is poor.

### 4.4. Comparison of Poisson’s Ratio between Experimental and Duncan Zhang’s E-v Model

The measured test ratio is the tangent Poisson’s ratio $\nu =\frac{d\varepsilon_{r}}{d\varepsilon_{a}}$. The test body curve has two points A ($\varepsilon_{a}{}_{1}$, $\varepsilon_{r1}$), B ($\varepsilon_{a2}$, $\varepsilon_{r2}$). The tangent Poisson’s ratio of middle point C ($\varepsilon_{a3}$, $\varepsilon_{r3}$) between A and B is $\nu_{c}=\frac{d\varepsilon_{r}}{d\varepsilon_{a}}=\frac{\Delta \varepsilon_{r}}{\Delta \varepsilon_{a}}=\frac{\varepsilon_{r2}-\varepsilon_{r1}}{\varepsilon_{a2}-\varepsilon_{a1}}$. On the stress–strain curve, the corresponding stresses at two points A and B are $\sigma_{m1}$, $\sigma_{m2}$, $\sigma_{m3}=\frac{\sigma_{m1}+\sigma_{m2}}{2}$, because the Level of stress $S=\frac{\sigma_{m}}{\sigma_{Max}}$, $\sigma_{Max}$ is the maximum value of the stress–strain curve. The measured experimental Poisson’s ratios under different stress levels are obtained successively and compared with the Poisson’s ratios calculated by Duncan Zhang’s E-v model.

**Figure 7:**
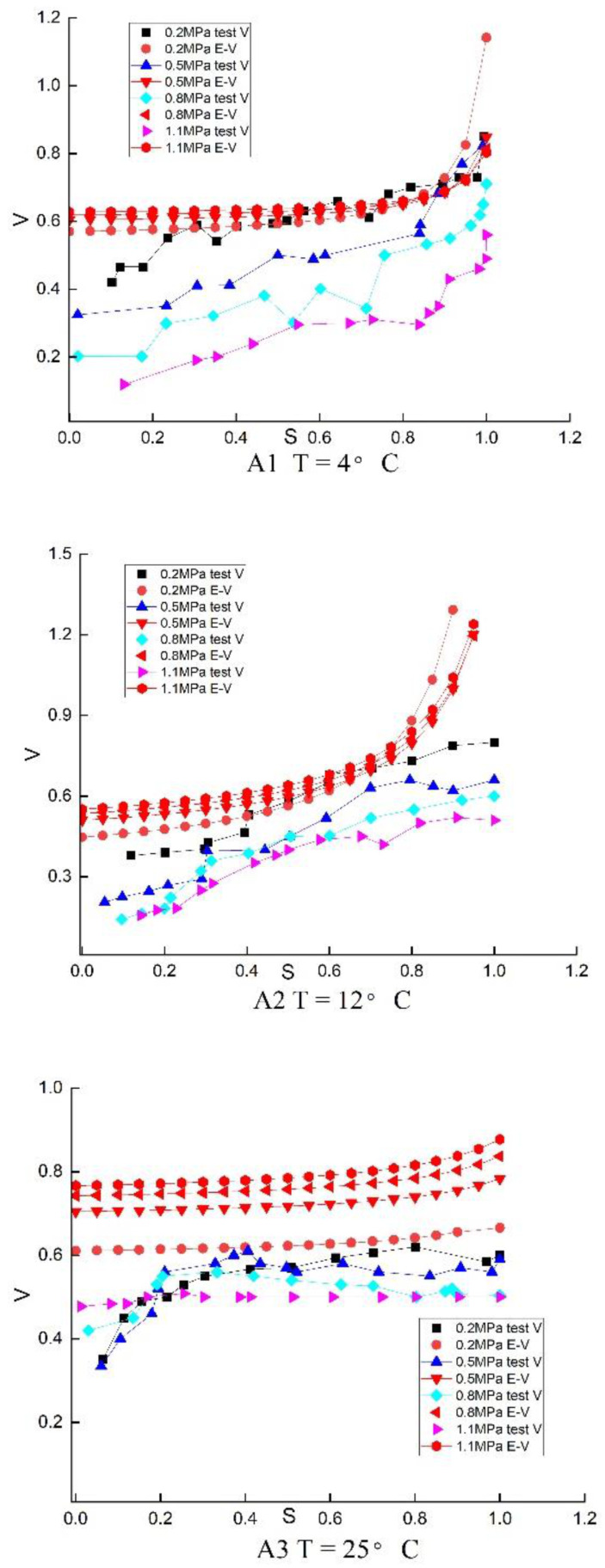
Poisson’s ratio of experimental and Duncan-Zhang E-v model.

It can be seen in Figure 7 that at 4 and 12 °C, Poisson’s ratio of Duncan Zhang’s E-v model increases with the stress level in a positive correlation. When the stress level is same, the confining pressure and Poisson’s ratio increases. Both of them are more than 0.5, which indicates that the volume is dilatant. The larger the confining pressure, the larger of dilatancy. However, different from Poisson’s ratio of Duncan Zhang’s E-v model, the larger the confining pressure, the smaller the test measured Poisson’s ratio. The test-measured Poisson’s ratio also increases with the stress level in a positive correlation. However, when the stress level is the same, the larger the confining pressure and the smaller the Poisson’s ratio. The Poisson’s ratio is less than 0.5 at a low-stress level and greater than 0.5 at a high-stress level, which indicates an initial volume shrinkage and then dilatation. 

In addition, it can be seen that except for the low confining pressure of 0.2 MPa, Poisson’s ratio of Duncan Zhang’s E-v model is larger than that of the test measured, and the gap between the two decreases with the increase of stress level. This indicates that the Poisson’s ratio of Duncan Zhang’s E-v model is poor at predicting the test-measured Poisson’s ratio when the stress level is low. At the same time, it is fit at high-stress levels. Since the Poisson’s ratio of Duncan Zhang’s E-v was greater than 0.5, but the Poisson’s ratio of the test is less than 0.5 at a low level and is greater than 0.5 at a high-stress level, there is a large gap between the volume of Ducan Zhang’s E-v model and test. So the fitting effect of Ducan Zhang’s E-v model to the test volume was poor, which was consistent with the conclusion of Section 4.3.

It can be concluded from Figure 7 that, different from the low-temperature condition, when the temperature is 25 °C, the measured Poisson’s ratio is more than 0.5 at the low-stress level. This indicates that recycled asphalt concrete has no shrinkage, only dilatancy at high temperatures. This indicates that the shear shrinkage of recycled asphalt concrete becomes weaker, and the dilatancy becomes stronger at high temperatures. In addition, because Duncan Zhang’s E-v model is a hardening type, Poisson’s ratio can be calculated until the max stress level. However, for the softening test curves, after reaching the max stress level, the stress level will decrease and also have Poisson’s ratio. So, Poisson’s ratio after the max stress level cannot be calculated by Duncan Zhang’s E-v model.

In summary, the differences between Poisson’s ratio of Duncan Zhang’s E-v model and the test-measured Poisson’s ratio are the reason for its poor fitting effect on the volumetric curve.

## 5. Conclusions

1. The shear strength parameters of recycled asphalt concrete at different temperatures have a great difference. The value of c decreases with the increase of temperature, while the value of φ increases. Therefore, the influence of temperature on the shear strength parameters of recycled asphalt concrete is particularly worth paying attention to. In the future constitutive model parameters, temperature as an influencing factor cannot be ignored. It is necessary to establish a temperature-related constitutive model, which will make the calculation of dam deformation and stability more realistic.
2. The stress–strain relationship of reclaimed asphalt concrete shows softening characteristics at low temperatures and confining pressure. It gradually evolves into a hardening type with the increase in temperature and confining pressure. The bulk transformation of reclaimed asphalt concrete is mainly shear contraction but followed by dilatancy. The dilatancy characteristics become more obvious at low temperatures and low confining pressure. With the increase in temperature and confining pressure, the dilatancy characteristics will weaken.
3. At low confining pressure, the fitting effect of Duncan Zhang’s E-v model on the stress–strain relation of recycled concrete is poor but better at high confining pressure. However, the fitting effect on the volume variation of asphalt concrete is not ideal and generally larger. The test curve consists of two parts: shear contraction and dilatancy, while the fitting curve is only dilatancy.
4. Under the same stress level, Poisson’s ratio of Duncan Zhang’s E-v model increases with the increase of confining pressure, while the measured Poisson’s ratio decreases. Poisson’s ratio calculated by Duncan Zhang’s E-v model is greater than 0.5, while the measured Poisson’s ratio is less than 0.5 at a low-stress level, and Poisson’s ratio is greater than 0.5 after reaching a certain stress level.

## Figures and Tables

**Table 1:** Test scheme.

Sample Name	Sample Density (g/cm^3^)	Test Temperature (°C)	Confine Pressure (MPa)	Rate of Loading Rate (mm/min)
A1	2.45	4	0.2, 0.5, 0.8, 1.1	0.5
A2	2.46	12	0.2, 0.5, 0.8, 1.1	0.5
A3	2.44	25	0.2, 0.5, 0.8, 1.1	0.5

**Table 2:** Duncan Zhang E-v model parameters.

Sample Name	c	φ	K	n	$R_{f}$	G	D	F
A1	1.05	24.3	1672	0.54	0.93	0.57	1.03	−0.06
A2	0.51	27.3	500	0.44	0.79	0.45	3.57	−0.10
A3	0.24	32.5	653	0.36	0.78	0.61	0.67	−0.16

## Data Availability

Not applicable.
